# Effect of SARS-CoV-2 infection on asthma patients

**DOI:** 10.3389/fmed.2022.928637

**Published:** 2022-08-02

**Authors:** Xin-yu Li, Jing-bing Wang, Hong-bang An, Ming-zhe Wen, Jian-xiong You, Xi-tao Yang

**Affiliations:** ^1^Department of Interventional Therapy, Multidisciplinary Team of Vascular Anomalies, Shanghai Ninth People’s Hospital, Shanghai Jiao Tong University, Shanghai, China; ^2^Department of Neurosurgery, Shanghai Ninth People’s Hospital, Shanghai Jiao Tong University, Shanghai, China; ^3^Affiliated Hospital of Weifang Medical University, School of Clinical Medicine, Weifang Medical University, Weifang, China

**Keywords:** SARS-CoV-2, asthma, COVID-19, differentially expressed genes, hub genes, drug molecules

## Abstract

**Background:**

SARS-CoV-2 causes coronavirus disease 2019 (COVID-19), a new coronavirus pneumonia, and containing such an international pandemic catastrophe remains exceedingly difficult. Asthma is a severe chronic inflammatory airway disease that is becoming more common around the world. However, the link between asthma and COVID-19 remains unknown. Through bioinformatics analysis, this study attempted to understand the molecular pathways and discover potential medicines for treating COVID-19 and asthma.

**Methods:**

To investigate the relationship between SARS-CoV-2 and asthma patients, a transcriptome analysis was used to discover shared pathways and molecular signatures in asthma and COVID-19. Here, two RNA-seq data (GSE147507 and GSE74986) from the Gene Expression Omnibus were used to detect differentially expressed genes (DEGs) in asthma and COVID-19 patients to find the shared pathways and the potential drug candidates.

**Results:**

There were 66 DEGs in all that were classified as common DEGs. Using a protein-protein interaction (PPI) network created using various bioinformatics techniques, five hub genes were found. We found that asthma has some shared links with the progression of COVID-19. Additionally, protein-drug interactions with common DEGs were also identified in the datasets.

**Conclusion:**

We investigated possible links between COVID-19 and asthma using bioinformatics databases, which might be useful in treating COVID-19 patients. More studies on populations affected by these diseases are needed to elucidate the molecular mechanism behind their association.

## Introduction

**FIGURE 1:**
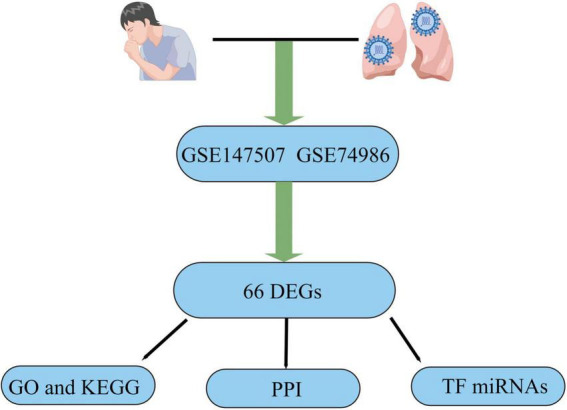
Article flow diagram.

SARS-CoV-2 is a serious infectious illness that risks people’s health worldwide (1). Fever, cough, and dyspnea are the most common clinical symptoms of COVID-19 infection. There are also rare cases with unusual symptoms, such as diarrhea, headaches, and a loss of smell (2). Although most patients have mild symptoms, some develop severe pneumonia and multi-organ failure and eventually die (3). COVID-19 spreads primarily through the air and touch, and elderly individuals with weakened immune systems and chronic diseases are more vulnerable to infection and have a higher fatality rate than healthy young people (4). Recurrent episodes of wheezing, shortness of breath, chest tightness, and coughing are among the clinical signs of asthma, which is often linked with high rates of morbidity and death (5, 6). Airway remodeling is an essential feature of asthma, manifested by structural changes in the airways, such as basement membrane thickening, bronchial fibrosis, airway smooth muscle cell hyperplasia, and hypertrophy (6–8). Among COVID-19 patients, uncontrolled asthma was identified as contributing factor to mortality, and patients also had more severe asthma manifestations during the pandemic (9, 10). Therefore, considering the current COVID-19 epidemic and the high prevalence of asthma, the study results are timely. To determine the biological connection between asthma and COVID-19, this study examined two datasets. GSE147507 and GSE74986 were for COVID-19 and asthma, respectively, and were obtained from the Gene Expression Omnibus (GEO) database. Differentially expressed genes (DEGs) were identified for the datasets, and the common DEGs for both diseases were obtained. Following research, including pathway analysis and enrichment analysis, were conducted based on the common DEGs to better understand the biological processes of genome-wide expression studies. Finally, the potential drugs were suggested. Figure 1 displays the entire workflow of our research.

## Materials and methods

### Identification of the common differentially expressed genes between asthma and COVID-19

The GEO database from the National Center for Biotechnology Information was used to download the gene expression profiles and to determine the common genetic interrelationships (11). The GEO accession number for the SARS-CoV-2 dataset was GSE147507, a transcriptional analysis of the response from COVID-19 lung biopsies to respiratory tract infections by extracting RNA sequences using a high-throughput sequencing Illumina NextSeq 500 platform (12). The asthma dataset was GSE74986, where the experimental platform was based on Agilent’s GPL6480 platform. The GSE74986 dataset contained 86 samples of cellular RNA from the bronchoalveolar lavage fluid of 86 subjects, including 74 samples from asthmatic subjects and 12 from normal subjects. The DEGs analysis was conducted using the R programming language. DEGs were identified using false discovery rate (FDR) ≤ 0.05, *P* < 0.05 and |log2 (fold change)| ≥ 1. Further, the Lim package in R software was used to analyze the DEGs, and the results were visualized through the R package. An online Venn analysis tool, jvenn, was used to obtain the common DEGs between GSE147507 and GSE74986.

### Construction of protein-protein interaction and hub gene screening analysis

Protein interactions are essential for most biological processes, and a PPI network is fundamental in understanding an organism’s functional and structural units. Thus, PPI networks provide a graph of cellular mechanisms (13). The online analysis website Search Tool for the Retrieval of Interacting Genes/Proteins (STRING)^1^ was used to construct the PPI network derived from the common DEGs and to display the functional and physical interactions between asthma and COVID-19, with a PPI combined score of >0.4 as a threshold condition. After constructing the PPI network of DEGs, the top five hub genes in the network were screened using the degree algorithm of the Cytoscape cytoHubba plugin (14). By examining the connectivity of the nodes in the PPI networks, the hub genes were determined. The cytoHubba plugin was used to distinguish the hub genes from the DEGs based on Maximal Clique Centrality (MCC) algorithm (15). Using the MCC approach of cytoHubba, the top five hub genes from the PPI network were identified.

### Gene ontology enrichment and Kyoto encyclopedia of genes and genomes pathway analysis

Functional enrichment analysis is often used in the genome and functional genome research to help researchers gain insights into biological significance, such as biological processes or chromosome positions related to different interrelated diseases (16, 17). The Enrichr database^2^ integrates 35 databases and offers a wide range of user-friendly enrichment analysis arrangements and visualizations. The enrichment analysis was calculated in three ways: first, using the conventional Fisher test. Second, a modified Fisher test, i.e., ranking using the z-score. Third, combining Fisher’s *p*-value and z-score in a combined ranking, i.e., the combined score. Fisher’s exact test is a statistical significance test. In this study, the gene ontology (GO) of DEGs and Kyoto encyclopedia of genes and genomes (KEGG) were analyzed by the Enrichr database. *P*-values < 0.05 were considered a standard metric for quantifying the above-listed pathways.

### Recognition of the transcription factors and miRNAs

Transcription factors (TFs) are proteins that bind to specific DNA sequences. In a disease condition, TFs control the expression of genes by interacting with their target genes during the transcription phase (18). The interaction network between DEGs-TFs was constructed using the JASPAR database^3^ built-in NetworkAnalyst. JASPAR is the most comprehensive publicly available database for transcription factor-DNA binding site motifs. The data in the JASPAR database are rigorously selected for their experimental validity, identified and matched by computer-aided software, and annotated by biological means (19, 20). The data is loaded for reanalysis into NetworkAnalyst, a visual analysis platform for comprehensive gene expression analysis (21). The Tarbase and mirTarbase databases were used to build a gene-miRNAs interaction network through NetworkAnalyst (22–24). In Cytoscape, a gene regulatory network containing DEGs, TFs, and miRNAs was built. This tool screens the top miRNAs with a high degree of biological function and characterization to derive valid biological hypotheses.

### Screening of the target drug candidates

The Drug Response Gene Signature Database (DSigDB), which incorporates drug response microarray data from public databases and scientific literature, is accessible via the online enrichment analysis tool Enrichr. It also provides drug and target information by screening the top 500 up-and down-regulated genes for drug signatures to create a DSigDB (25, 26). The current DSigDB has around 1,300 medicines, 7,000 microarrays, and 800 targets, allowing computational drug repositioning to be used to design innovative drugs targeting key genes (26). In this study, DSigDB was used to screen candidate drugs interacting with the hub genes to provide a reference for targeted therapy of diseases. *P* < 0.05 was considered statistically significant.

### Gene-disease association of differentially expressed genes

DisGeNET^4^ is one of the largest and most comprehensive human gene-disease association repositories, synchronizing relationships from several sources and featuring various biomedical aspects of diseases (27, 28). DisGeNET now has over 24,000 illnesses, 17,000 genes, and 117,000 genomic variations. NetworkAnalyst was also utilized to investigate gene-disease connections in order to find illnesses and chronic issues linked to common DEGs.

## Results

### Identification of the common differentially expressed genes between asthma and COVID-19

**FIGURE 2:**
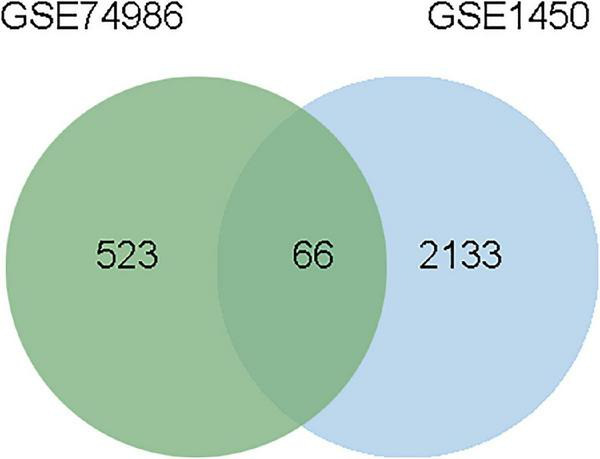
This study incorporates asthma (GSE74986), and SARS-CoV-2 (GSE147507). This integrated analysis revealed 66 common DEGs are shared among SARS-CoV-2 and asthma.

To identify the common DEGs between COVID-19 and asthma patients, we extracted all the all significant DEGs with FDR ≤ 0.05 and |log2 (fold change)| ≥ 1 were extracted. In the GSE74986 dataset, 589 DEGs were identified, of which 43 were highly expressed, and 546 were lowly expressed genes. In the GSE147507 dataset, 1,184 DEGs were identified, of which 293 were highly expressed, and 891 were lowly expressed genes. We identified 66 common DEGs from asthma and SARS-CoV-2 datasets (Figure 2 and Table 1). A Venn diagram was generated using jvenn web.^5^ This common gene set was used for further experiments. A Venn diagram was generated using jvenn web (see text footnote 5) as previously described (28). This common gene set was used for further experiments.

**TABLE 1:** Common genes of asthma and COVID-19 (*n* = 66).

NEAT1	CLK1	HBP1	METTL18	CLK4	SLC2A3	RNF6
IFRD1	JMJD1C	EREG	CD300LF	ADNP2	KCNAB1	ZNF761
CXCL2	SESN1	CCNG1	NBPF14	GPCPD1	RBPJ	ZNF12
SAMSN1	WFDC2	ERRFI1	NFIL3	CBWD5	ZNF654	FAM126B
NR4A3	CLEC7A	ZBTB1	VMP1	CCNT2	TIMP1	CD55
MALAT1	FCGR3B	ZNF302	BTAF1	SCAND1	MMP25	STON1-GTF2A1L
MAP3K1	ARMCX3	RCHY1	ZNF267	MOAP1	CHD1	
NFE2	CMAHP	ZNF181	HNRNPH2	NECAP1	RANBP6	
CCNL1	BTK	TSC22D1	CDKN1B	APBB1IP	CFP	

### Gene ontology and pathway enrichment analysis

**FIGURE 3:**
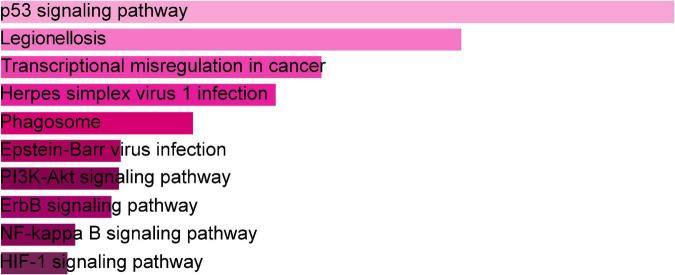
The bar graphs of pathway enrichment analysis of shared DEGs among SARS-CoV-2, asthma performed by the Enricher online tool.

The online tool Enrichr (see text footnote 2) was used for gene enrichment analysis and to determine the biological significance, and enrichment pathways of the DEGs shared in this study (29). The GO database is a comprehensive resource of computable knowledge on the function of genes and gene products (30). GO annotations in biological process (BP), molecular function (MF), and cellular component (CC) were retrieved from the GO database (31). Supplementary Table 1 displays the top 10 enriched GO categories of BP, CC, and MF. For BP, the genes were mainly enriched in regulation of transcription, DNA-templated, regulation of RNA metabolic process, and positive regulation of smooth muscle cell proliferation. For CC, genes were mainly enriched in the nucleus. For MF analysis, the module showed predominant enrichment in cyclin-dependent protein serine/threonine kinase regulator activity. According to the KEGG analysis, the DEGs were primarily abundant in the p53 signaling pathway, legionellosis, transcriptional misregulation in cancer, and PI3K-Akt signaling pathway. Figure 3 represents the pathway enrichment analysis in the bar graphs. In addition, GO, KEGG pathway analysis, Gene counts, and gene symbols were shown in Supplementary Tables 2, 3, respectively.

### Classification of the hub genes and submodules

**FIGURE 4:**
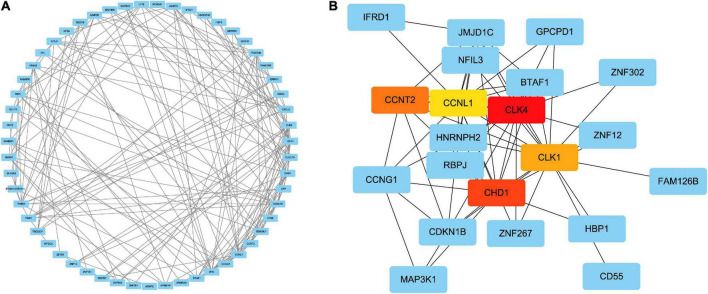
**(A)** PPI network of common DEGs among SARS-CoV-2 and asthma. In the figure, the circle nodes represent DEGs and edges represent the interactions between nodes. The PPI network has 64 nodes and 166 edges. **(B)** Determination of hub genes from the PPI network by using the Cytohubba plugin in Cytoscape. The latest MCC procedure of Cytohubba plugin was pursued to obtain hub genes. Here, the red nodes indicate the highlighted top five hub genes and their interactions with other molecules.

To forecast the contacts and adhesion routes of the common DEGs, we investigated the PPI network created by STRING and displayed it in Cytoscape. The PPI network of the DEGs contained 64 nodes and 166 edges, where the most tangled nodes were the hub genes (Figure 4A). The top five hub genes with the highest MCC scores were determined using Cytoscape’s cytoHubba plugin. The hub genes were *CLK4*, *CLK1*, *CHD1*, *CCNL1*, and *CCNT2* (Figure 4B). In addition, we conducted a ROC analysis for asthma and COVID-19 (Supplementary Figure 1). Among these five hub genes in the COVID-19 cohort, all had AUC values larger than 0.65, with 3 hub genes with AUC values larger than 0.7. In the asthma cohort, the AUC values of all hub genes were greater than 0.6. These hub genes could serve as potential biomarkers and might lead to new therapeutic strategies.

### Determination of regulatory networks

**FIGURE 5:**
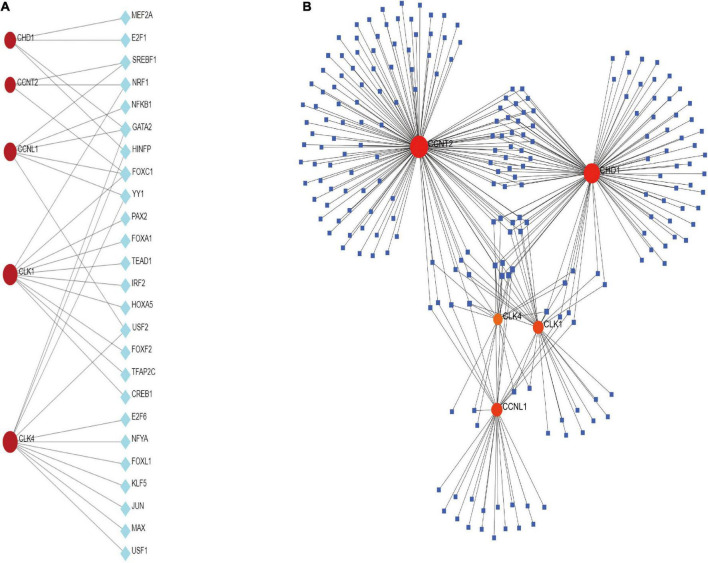
**(A)** The cohesive regulatory interaction network of DEG–TFs obtained from the Network Analyst. **(B)** The interconnected regulatory interaction network of DEGs–miRNAs.

Transcription factors and miRNAs are critical regulators of gene expression at the transcriptional and post-transcriptional levels (32). TFs and miRNAs can work together to control the same genes. TFs regulate the transcription of miRNAs, and a common set of target lncRNAs are regulated by both TFs and miRNAs. TFs and miRNAs together make up a complex regulatory network (33). From TFs and miRNAs gene interaction network analysis, we established 26 TFs and 1,123 post-transcriptional (miRNAs) regulatory signatures regulated by more than one common DEG, indicating a strong interaction between them (Figure 5). We also constructed and analyzed regulatory networks of target TF and target miRNA genes, including topology tables (Supplementary Tables 4, 5).

### Gene-disease associations

**FIGURE 6:**
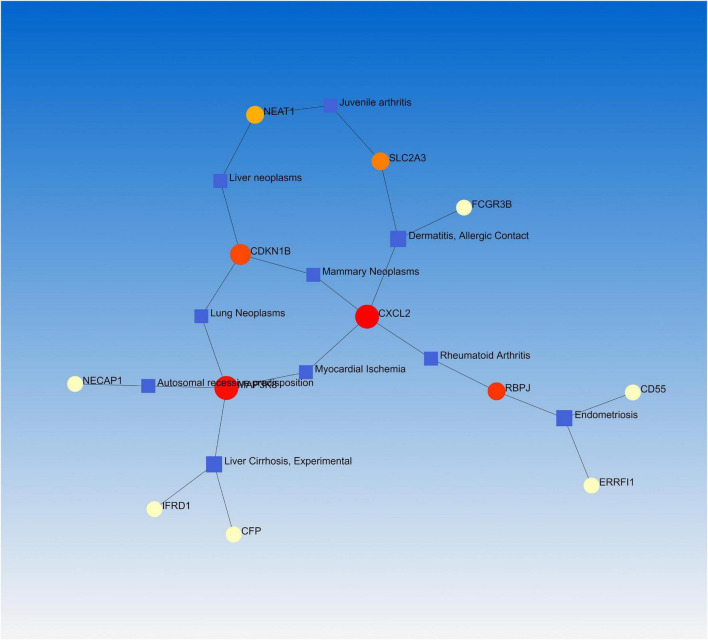
The gene-disease association network represents diseases associated with mutual DEGs.

Numerous studies have demonstrated that a common or related gene set is involved in similar or related diseases, allowing us to uncover the relationships between genes and diseases (34, 35). NetworkAnalyst was used to analyze the gene-disease association. We found that endometriosis, dermatitis, allergic contact, rheumatoid arthritis, and juvenile arthritis were mainly associated with our reported hub genes (Figure 6).

### Identification of candidate drugs

The interaction between the drug and the protein is vital to understand the structural features recommended for receptor sensitivity. DSigDB contains the largest number of drugs/compound-related gene sets to date. According to the DSigDB database, drug molecules were suggested from the common DEGs. The top 10 chemical compounds were extracted based on their *p*-values (Table 2). Therefore, these drugs could potentially be used as common chemical compounds for treating the two diseases.

**TABLE 2:** List of the suggested drugs for COVID-19.

Term	*P*-value	Chemical formula	Structure
Mitoxantrone	2.19E-14	C_22_H_28_NO_4_O_6_	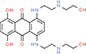
Anisomycin	7.06E-14	C_14_H_19_NO_4_	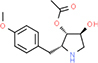
H-7	2.29E-13	C_14_H_17_N_3_O_2_S	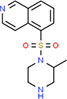
Staurosporine	2.02E-12	C_28_H_26_N_4_O_3_	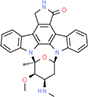
Doxorubicin	4.52E-12	C_27_H_29_NO_11_	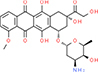
Ouabain	4.68E-12	C_29_H_44_O_12_	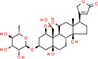
Daunorubicin	8.18E-12	C_27_H_29_NO_10_	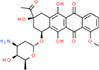
Puromycin	2.07E-11	C_22_H_29_N_7_O_5_	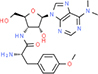
Digitoxigenin	7.73E-11	C_23_H_34_O_4_	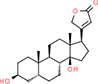
Alsterpaullone	1.12E-10	C_16_H_11_N_3_O_3_	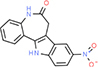

## Discussion

Mahmud’s work on the impact of SARS-CoV-2 infection in individuals with idiopathic pulmonary fibrosis and chronic obstructive lung disease inspired this investigation (28). We explored a unique perspective of the potential interaction between asthma and COVID-19. Our primary objective was to examine the association between asthma and COVID-19. As a chronic respiratory disease, asthma is characterized by an inherent chronic airway inflammation involving multiple immune cells (eosinophils and mast cells), usually accompanied by increased airway reactivity. Often, acute exacerbations of asthma after pneumonia not only increase patient visits and hospital admissions but also lead to adverse clinical outcomes, such as respiratory failure or death. Additionally, patients with severe asthma take high doses of systemic corticosteroids for long periods, leading to systemic diseases (e.g., hypertension and diabetes) and thus increasing mortality after pneumonia.

The COVID-19 infection is a multi-organ disease characterized by the activation of cellular and innate immunity, often resulting in lung inflammation (36). Hence, COVID-19 infection is a high-risk factor for asthma exacerbations. Meanwhile, studies show that virus-associated respiratory infections are particularly severe in patients with asthma. Some studies also suggest that adults with asthma are at a higher risk of developing severe COVID-19 (37). Furthermore, the Centers for Disease Control and Prevention has identified asthma as a possible risk factor for COVID-19 after treating asthmatic patients with severe viral infections (38). Previous studies have shown that in addition to age, gender, smoking, and several co-morbidities (e.g., obesity, type 2 diabetes, hypertension, etc.), ongoing medication might also be strongly associated with the severity of COVID-19 and adverse clinical outcomes (39). Usually, the prognosis is poor when the co-morbidity is a risk factor, especially in patients with COPD. However, the association of asthma, another major chronic airway disease, with the clinical outcomes of COVID-19 is still unclear.

Here, we developed a general co-expression network-based approach to analyze both gene sets in the microarray data that might act as potential biomarkers of COVID-19. Together, they provide critical information about the disease progression and the development of drug resistance and contribute to discovering new therapeutic approaches. The investigation of asthma and COVID-19 transcriptomics indicated that the common 66 DEGs had comparable expression patterns in both illnesses. Based on the *P*-values, these DEGs were analyzed using the GO pathway analysis function to give insights into the biological relevance in the etiology of asthma and COVID-19.

Gene ontology is a consortium-based dataset that provides information on the functions of genes. In BP, regulation of transcription and DNA-templated were among the top GO terms. In MF, cyclin-dependent protein serine/threonine kinase regulator activity and protein kinase regulator activity were the top two GO pathways. Under CC, nucleus and intracellular membrane-bounded organelle were the top two GO terms. To establish a comparable pathway for asthma and COVID-19, the KEGG pathway of the 66 shared DEGs was found. The top KEGG human pathway includes the p53 signaling pathway, Legionnaires’ disease, transcriptional misregulation in cancer, herpes simplex virus 1 infection, and phagosome. The host protease TM-PRSS2 cleaves the S protein on the surface of SARS-CoV-2 after the S protein binds to ACE2, prompting virus entry into the host cell (40). Earlier studies reported that p53 inhibits coronavirus replication by degrading ACE2 in humans. Deleting the p53 binding site in porcine kidney cells increases ACE2 promoter activity (41).

A PPI network of DEGs was constructed using the STRING database to uncover the potential association between DEGs. Finding hub genes from common DEGs is of utmost importance because the discovery of drug molecules is primarily dependent upon them. We found five hub proteins (*CLK4*, *CLK1*, *CHD1*, *CCNL1*, and *CCNT2*) involved in these diseases. Here, the cutoff of the topological metric for the hub genes was 10 (degrees). The CLK family consists of four isoforms: *CLK1*, *CLK2*, *CLK3*, and *CLK4* (42). *CLK1* and *CLK4* are very similar in their amino-acid sequence (42). *CLK1* is vital in splicing the H1N1 influenza virus M2 gene and is a critical anti-influenza target (43). *CHD1* is composed of 1,710 amino acids and plays an essential role in transcriptional regulation and development (44). *CHD1* controls chromatin structural regions through chromatin accessibility and nucleosome depolymerization and plays a role in the development and progression of prostate cancer (45). *CCNL1* is a member of the cell cycle protein family. It is involved in the precursor mRNA shearing and RNA processing and is associated with the action of cell cycle protein-dependent kinases (46). Additionally, *CCNL1* may contribute to cell survival during cellular stress, such as cold stimulation (47). *CCNT2* is a cyclin involved in the cell cycle and RNA transcription (48). Thus, the identified hub genes could be potential biomarkers and new drug targets. However, more studies are needed to explore the biological insights of COVID-19.

Understanding how miRNAs downregulate target genes and TFs regulate miRNAs during post-transcriptional regulations is critical. Hence, we investigated TFs and miRNAs that potentially regulate the identified DEGs. The identified TFs are associated with different functions, such as cell development and cancer. Further, some miRNAs are involved in lung cancer (hsa-mir-335-5p), infectious diseases (hsa-mir-16-5p), and hematological malignancies (hsa-miR-155-5p) (49). Most miRNAs are associated with cancerous tissues and cause different cancers in the human body. We performed a gene-disease analysis to predict the association of important DEGs with different diseases. The results showed that COVID-19 is associated with various diseases, including lung tumors, allergic diseases, liver, skin, and joints diseases. People with lung diseases are at a higher risk of COVID-19 (50). Our study also identified several genes associated with lung diseases, such as lung cancer. Our network analysis also found some dermatitis, such as allergic contact. Recently, skin diseases in patients with COVID-19 infection were reported in Italy. Side effects of COVID-19 infection include itchy skin. The liver damage from COVID-19 is directly related to the cytopathic effects of the virus, derangement of the immune response, and drug damage. In patients with rheumatoid arthritis, various immune cells are abnormally activated, causing immune dysfunction and making the body susceptible to pathogenic microbial infections, including COVID-19 (51).

Although no standard drugs or vaccines are available for COVID-19, several chemicals and drugs are being investigated as treatment agents (52). We have identified a variety of drugs as potential therapeutic agents. Researchers have found that ouabain is resistant to SARS-CoV-2 and inhibits over 99% of SARS-CoV-2 replication, resulting in suppression of the virus in the post-life cycle entry phase (53). Ouabain might serve as an alternative therapy to COVID-19 and has potential additional therapeutic benefits for patients with cardiovascular disease (53). Fasudil, a selective ROCK inhibitor, plays various roles with T cells (54). Paclitaxel, an anti-tumor drug, can increase the levels of methylglyoxal in cells, providing a powerful reason to repurpose Doxorubicin and Paclitaxel for COVID-19 treatment (55). Daunorubicin is a DNA topoisomerase II, known to disrupt either the catalytic cycle of topoisomerase II (catalytic inhibitors) or stabilize the topoisomerase II–DNA transient cleavage complex (topoisomerase II poisons). M. Bouachrine study suggests Digitoxigenin as an inhibitor against SARS-CoV-2 (56). Mitoxantrone is used in diseases related to anti-malignant disorders, including leukemia, lymphoma, and lung cancer (57). In addition to respiratory tumors, COVID-19 infections involve different tumor sites; therefore, all these drugs could potentially treat COVID-19 infections (58). However, several potential limitations of the study must be acknowledged. No functional and mechanistic experiments were conducted to validate the GO and KEGG results. In addition, we did not perform mechanistic experiments to confirm the results from computational analysis. Therefore, more prospective studies are needed to confirm our results.

## Conclusion

Our study used transcriptome analysis to summarize the relationship between asthma and COVID-19. We obtained DEGs from GEO datasets, identified the common genes, and found an association between asthma and SARS-CoV-2. Further, a PPI network was generated using the 66 common genes, and the five most essential hub genes were identified from the PPI network. All hub genes play important roles in different types of respiratory diseases. Therefore, the genes we identified could potentially be a new therapeutic target for developing the COVID-19 vaccine.

## Data availability statement

The datasets presented in this study can be found in online repositories. The names of the repository/repositories and accession number(s) can be found in the article/Supplementary material.

## Author contributions

All authors made substantial contributions to conception and design, acquisition of data, or analysis and interpretation of data; took part in drafting the article or revising it critically for important intellectual content; agreed to submit to the current journal; gave final approval of the version to be published; and agreed to be accountable for all aspects of the work.

## Footnotes

1
https://string-db.org/

2
http://amp.pharm.mssm.edu/Enrichr/

3
http://jaspar.genereg.net

4
https://www.disgenet.org/

5
http://jvenn.toulouse.inra.fr/app/example.html

## Supplementary material

Supplementary Figure 1 Validation of the hub gene by ROC analysis. **(A)** was ROC analysis of COVID-19 cohort; **(B)** was ROC analysis of asthma cohort.

Supplementary Table 1 Ontological analysis of common differentially expressed genes (DEGs) among COVID-19 and asthma.

Supplementary Table 2 Gene ontology (GO) pathway.

Supplementary Table 3 Kyoto encyclopedia of genes and genomes (KEGG) pathway.

Supplementary Table 4 Transcription factor (TF)-Gene topology table.

Supplementary Table 5 MiRNA-gene topology table.
