# Supplementary figures and images for: Effect of SARS-CoV-2 infection on asthma patients
Source: Front Med (Lausanne). 2022 Aug 2;9:928637. doi: 10.3389/fmed.2022.928637 (PMC9378965; doi:10.3389/fmed.2022.928637)

SARS-CoV-2

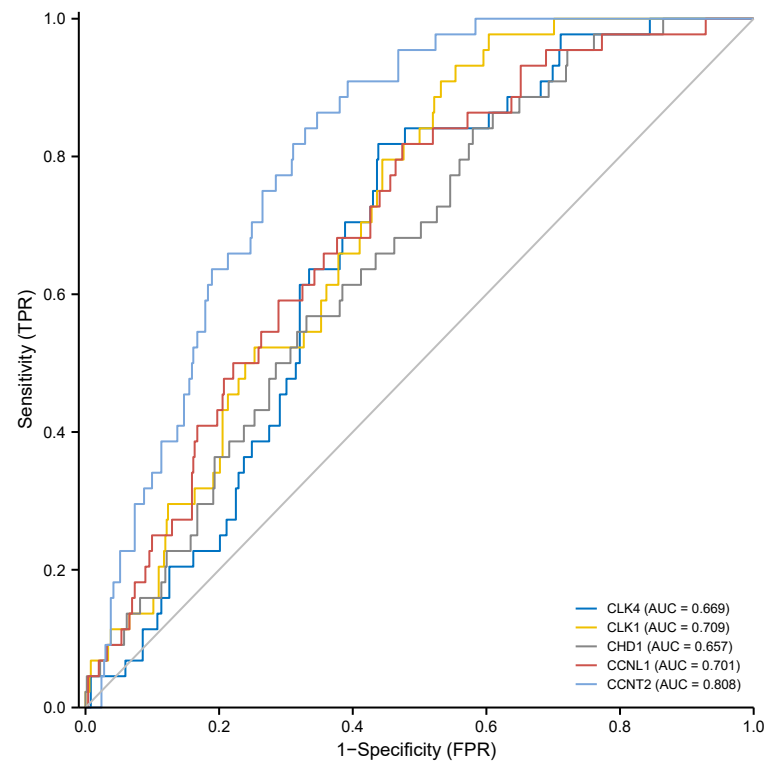

Asthma

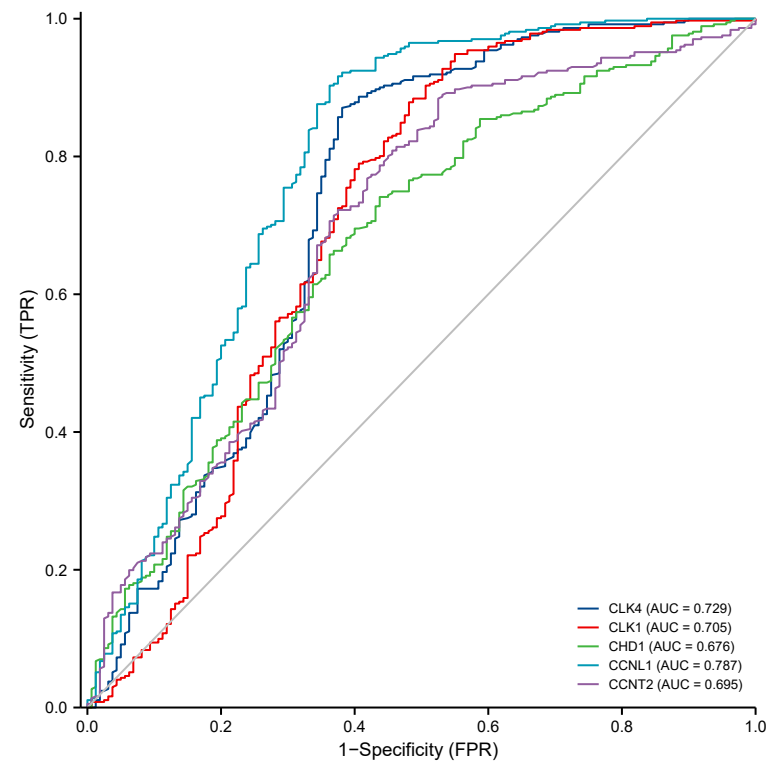

Supplement: Supplementary Figure 1 — Validation of the hub gene by ROC analysis. (A) was ROC analysis of COVID-19 cohort; (B) was ROC analysis of asthma cohort. [file Data_Sheet_1.PDF]
